# *Cinnamomum burmannii* Essential Oil as a Promising Antimicrobial Agent Against Cutaneous Pathogens: Mechanistic Insights into Its Anti-*Malassezia furfur* Activity

**DOI:** 10.3390/microorganisms13061241

**Published:** 2025-05-28

**Authors:** Wenwen Wang, Shuizhu Cai, Ying Wang, Yanhui Tan, Jing Xu, Ping Xiong

**Affiliations:** Department of Pharmaceutical Engineering, South China Agricultural University, Guangzhou 510642, China; wellwinwen@163.com (W.W.); iamcaishuizhu@163.com (S.C.); w18824714168@163.com (Y.W.); 15294771418@163.com (Y.T.); 17898488256@163.com (J.X.)

**Keywords:** *Malassezia furfur*, *Cinnamomum burmannii*, antifungal activity, mechanism of action, ergosterol

## Abstract

This study investigated the chemical composition, antibacterial activity and antifungal mechanisms of *Cinnamomum burmannii* essential oil (CBEO) obtained from leaves and branches through pilot-scale steam molecular distillation after D-borneol crystallization, focusing on its inhibitory effects against *Malassezia furfur* (*M. furfur*). GC-MS analysis identified 78 chemical constituents in CBEO, with the monoterpenoid D-borneol predominating. CBEO exhibited potent antifungal activity against *M. furfur*, with MIC and MFC values of 0.88 mg/mL and 1.75 mg/mL, respectively. Synergistic effects were observed when combined with ketoconazole (FICI = 0.5). At 2 × MIC concentration, CBEO suppressed 85.6% of biofilm formation (*p* < 0.01) as determined by crystal violet assay. SEM imaging revealed that CBEO treatment induced the formation of surface invaginations and pore structures on fungal cells. Quantitative detection of intracellular protein, nucleic acid, and ion leakage levels confirmed CBEO enhanced membrane permeability, resulting in cytoplasmic content leakage. Ergosterol binding assays confirmed cell membrane disruption (8-fold MIC increase), while UPLC quantification demonstrated dose-dependent suppression of ergosterol synthesis. Correspondingly, squalene epoxidase (SE) activity was significantly inhibited in treated cells. These findings systematically elucidate CBEO’s anti-*M. furfur* mechanisms, highlighting its potential as a natural antifungal agent for cosmeceutical applications.

## 1. Introduction

The cutaneous microbiome, the largest microbial ecosystem in humans, plays a pivotal role in maintaining skin homeostasis through intricate ecological interactions [1,2]. Among its constituents, *Malassezia furfur*—a lipid-dependent basidiomycetous yeast colonizing the stratum corneum—has drawn particular attention due to its pathophysiological implications [3]. The genus *Malassezia* is taxonomically classified under the phylum *Basidiomycota*, subphylum *Ustilaginomycotina*, class *Malasseziomycetes*, order *Malasseziales*, and family *Malasseziaceae* [4]. To date, 21 species have been identified, with 14 documented as human-associated pathogens [5]. *Malassezia* predominantly colonizes sebum-rich regions, including the scalp, face, neck, upper chest, and cutaneous folds [6]. Through the secretion of lipases (Lip1/Lip2) and phospholipase (Phl1), it hydrolyzes triglycerides into free fatty acids (FFAs), thereby enhancing its virulence [7,8]. Key metabolites such as oleic acid and arachidonic acid induce aberrant keratinocyte differentiation, leading to pathological epidermal manifestations, including parakeratosis, irregular keratinocyte membrane morphology, disruption of epidermal barrier integrity, and activation of inflammatory cascades. The lipophilic nature of *M. furfur* not only dictates its ecological niche but also correlates with dermatological disorders, including pityriasis versicolor (PV), *Malassezia* folliculitis (MF), and seborrheic dermatitis/dandruff (SD/D) [9]. Affecting approximately 3% of the global population, SD/D manifests as erythema, pruritus, and scaling, substantially impairing patients’ quality of life [10,11].

*Malassezia* spp., as eukaryotic organisms share significant similarities in metabolic pathways with host cells. The biological convergence between eukaryotic hosts and fungal pathogens has led to a primary focus in drug development on targeting unique structural and functional components specific to the pathogen [12]. Current therapeutic strategies against *M. furfur*-related SD/D primarily rely on azoles, zinc pyrithione (ZPT), keratolytic agents, etc. However, emerging concerns over azole-induced hepatorenal toxicity [13], microbial resistance [14], and the recent EU ban on ZPT due to carcinogenic risks [15] underscore the urgent need for safer antifungal alternatives. Natural products, particularly plant-derived essential oils with multifunctional activities, have thus gained momentum in dermatological research [16,17].

*Cinnamomum burmannii* (Nees & T. Nees) Blume (*Lauraceae*), an evergreen tree native to Southeast Asia, yields volatile oils rich in bioactive monoterpenes and sesquiterpenes. Essential oils extracted from *Cinnamomum burmannii* leaves have demonstrated anti-inflammatory [18], antimicrobial [19], and antioxidant properties [20] in previous studies. While existing investigations into the antimicrobial properties of *Cinnamomum burmannii* essential oil (CBEO) have predominantly focused on common bacterial and fungal pathogens such as *Staphylococcus aureus*, *Escherichia coli* [21] and *Aspergillus flavus* [22], its inhibitory effects against cutaneous mycobiota—particularly the lipophilic yeast *Malassezia furfur*—remain unelucidated.

Notably, no systematic study has yet characterized the antifungal mechanism of CBEO against *M. furfur*, representing a critical knowledge gap in dermatological phytotherapy research. We herein present the first evidence of CBEO’s anti-*Malassezia* activity and its synergistic interaction with ketoconazole against *M. furfur* while preliminarily exploring the mechanisms of action and molecular targets underlying CBEO’s inhibitory effects on *M. furfur*.

## 2. Materials and Methods

### 2.1. Microbial Strain and Culture Conditions

The standard strain *Malassezia furfur* ATCC 44344 was obtained from the Guangdong Microbial Culture Collection Center (GDMCC, Guangzhou, China).

*Malassezia furfur* strains were cultured and preserved under the GDMCC’s recommended conditions. The revival protocol comprised streaking cryopreserved stock cultures onto modified Dixon agar (M-Dixon; Hope Bio-Technology Co., Ltd., Qingdao, China) followed by aerobic incubation at 32 °C for 48 h. For experimental preparations, single colonies were inoculated into 100 mL of 2693-modified Dixon broth (Solarbio) and incubated at 32 °C with orbital shaking (120 rpm) for 24–30 h. Cell pellets were harvested by centrifugation (8000× *g*, 5 min, 4 °C), washed twice with sterile PBS (pH 7.4), and resuspended in fresh medium. The optical density of activated cultures was standardized to 0.5 McFarland units (1 × 10^6^ CFU/mL) using a densitometer (DEN-1B, Biosan). Freshly prepared inoculum was maintained at 4 °C and utilized within 4 h to ensure viability.

### 2.2. Preparation of Essential Oils Extract

**Figure 1:**
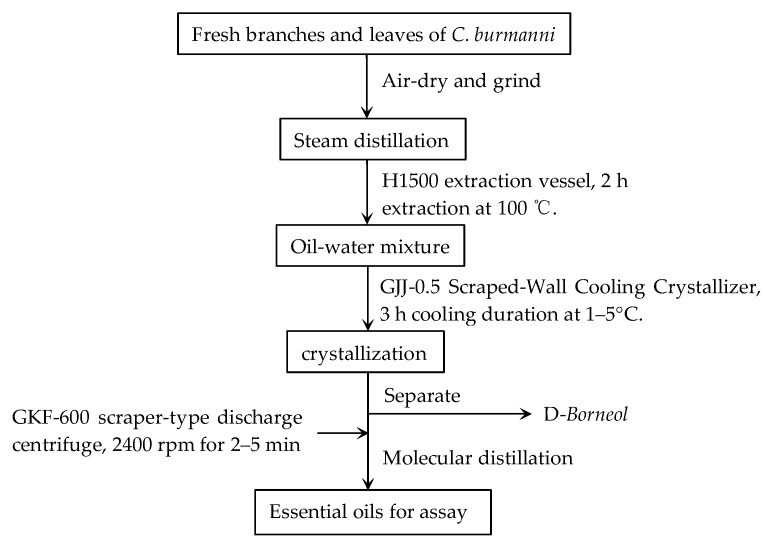
Extraction and process procedure.

The branches and leaves of *Cinnamomum burmanni* Blume were collected from Dazhe Town, Pingyuan County, Guangdong province. Fresh branches and leaves of *Cinnamomum burmannii* Blume were dried in the shade, processed into a rough powder, and then sifted through a 24-mesh sieve. The crude oils were extracted by water steam distillation and then dehydrated with anhydrous sodium sulfate to further vacuum suction filtration. Finally, *Cinnamomum burmannii* essential oils were separated from crude oils using molecular distillation technology. CBEO is a light yellow transparent oily liquid with a special flavor. The preparation procedure is as follows (Figure 1).

### 2.3. GC-MS Analysis of CBEO

*Cinnamomum burmannii* essential oil (0.1 g) was accurately weighed and dissolved in 20 mL of n-hexane. The mixture was vortexed, and the supernatant was filtered through a 0.22 μm membrane filter. The characteristic components of this volatile essential oil were identified using an Agilent 7890B Network GC system combined with an Agilent 5977B Inert MSD detector (quadruple) in the electron impact mode (70 eV). Chromatographic separation was achieved using a HP-5MS 5% Phenyl Methyl Siloxane column (300 × 0.25 mm, 0.25 µm) (Agilent, Santa Clara, CA, USA). Helium was used as carrier gas at a 2 mL/min flow rate without a split flow. The temperature conditions of GC were as follows: 80 °C for 1 min, rate of 15 °C/min from 80 °C increased to 250 °C and held for 5 min. The detector was held at 300. The mass spectrometer was bombarded with an electron impact ionization source, and the electron energy was 70 eV. The inlet temperature was 275 °C, and the ion source temperature was 230 °C. The emission current was 34.6 μA, with a 1392 V electron multiplier voltage. The mass scanning range was 33–450 *m*/*z*. The compounds were identified by comparing their Kovats indices (KI), GC retention times (authentic chemicals), NIST mass spectral search program (version 2.0, National Institute of Standards and Technology) and mass spectra of published data. For components isolated, the relative percentage amount in the essential oil was calculated according to the corresponding peak area of the total ion chromatogram and expressed as relative (percent) areas by normalization.

### 2.4. Chemical Stability of CBEO

To verify the chemical stability of CBEO under experimental conditions, this study conducted stability testing over a 180-day storage period at 40 °C prior to antimicrobial assays. Gas chromatography-mass spectrometry (GC-MS) was employed to monitor content variations of four characteristic components (α-terpineol, camphor, D-borneol, and eucalyptol) across three CBEO batches (Batch Nos.: 202107008, 202111005, 202204002). Samples were stored in a temperature-humidity chamber (40 °C ± 2 °C, 75% RH ± 5% RH) for 180 days. Triplicate samples from each batch were collected at 0, 30, 60, 90, 120, 150, and 180 days (*n* = 6 time points). Precisely weighed 0.5 g aliquots (analytical balance, ±0.0001 g) were dissolved in ethyl acetate (10 mL volumetric flask), vortex-mixed for 10 min, and filtered through 0.45 μm membranes. Utilized identical GC-MS parameters as described in Section 2.3.

### 2.5. Antifungal Activity of CBEO

#### 2.5.1. Determination of Minimum Inhibitory and Fungicidal Concentration Values

Determination of Minimum Inhibitory Concentration (MIC) and Minimum Fungicidal Concentration (MFC) values of CBEO against *M. furfur* via Microbroth Dilution Method [23]. *M. furfur* cultures in the logarithmic growth phase were adjusted to 0.5 McFarland standard and diluted with 0.85% NaCl to 1 × 10^6^ CFU/mL. The diluted suspension was dispensed into 96-well microplates, followed by the addition of gradient-diluted CBEO. Each well contained a mixture of 100 μL fungal suspension and 100 μL CBEO solution. The microplates were incubated at 32 °C for 48 h. The MIC was defined as the lowest concentration, showing no visible fungal growth macroscopically.

**Figure 2:**
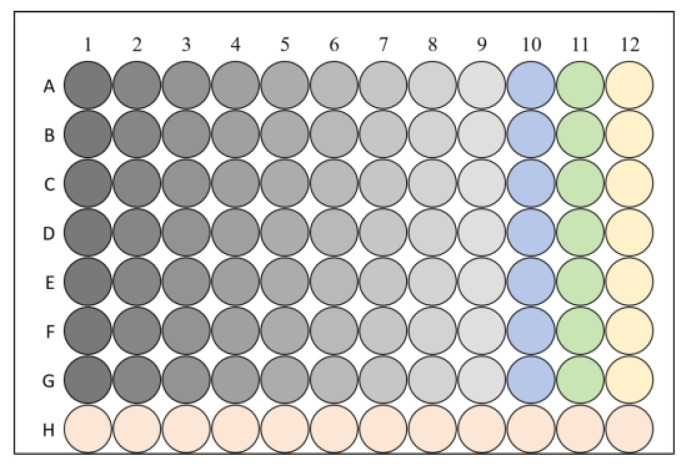
MIC plate layout. Wells of the same color represent biological replicate groups. A1–A9 (left to right) indicates descending concentrations of CBEO. A10–G10: Positive control group (ketoconazole). A11–G11: Negative control group (solvent: 10% PEG400). A12–G12: Growth control group (untreated *M. furfur* suspension). H1–H12: Sterile liquid medium (uninoculated).

The minimum fungicidal concentration was determined as the minimal drug concentration, achieving >99.9% of the initial inoculum’s killing rate. To assess MFC, 50 μL aliquots from wells with concentrations ≥ MIC were subcultured onto agar plates. After 48-h incubation at 32 °C, CBEO’s MFC was identified when colony counts were reduced to <0.1% of the original inoculum. The microplate layout is illustrated in Figure 2. The experiment was carried out with three replicates per treatment and repeated twice.

#### 2.5.2. Checkerboard Assay

The checkerboard microdilution assay, modified from the method described by Dehghan et al. [24], was employed to evaluate the fractional inhibitory concentration index (FICI) of CBEO and ketoconazole (KCZ). *M. furfur* suspensions were prepared as previously described. CBEO and KCZ were tested across a concentration range of 1/64 × MIC to 4 × MIC. The fractional inhibitory concentration (FIC) and fractional inhibitory concentration index (FICI) were calculated as follows, with all experiments performed in triplicate. FICI ≤ 0.5: Synergistic effect; 0.5 < FICI ≤ 4: Indifference; FICI ≥ 4: Antagonistic effect [25].

FIC = MIC_combination_/MIC_alone_ FICI = FIC_A_ + FIC_B_ (1)

### 2.6. Time-Kill Kinetics Analysis of CBEO Against M. furfur

The time-kill kinetics assay, modified from the protocol described by Blais et al. [26], was conducted to evaluate CBEO’s antimicrobial mode of action against *M. furfur*. Logarithmic-phase *M. furfur* cultures (0.5 McFarland) were treated with 1 × MIC, 2 × MIC, and fractional inhibitory concentration (FIC) combinations of CBEO with ketoconazole, followed by incubation in a 32 °C incubator. Over a 32-h period, samples were vortex-mixed every 2 h. At each interval, 100 μL aliquots were withdrawn from the fungal suspensions, subjected to serial two-fold dilution with 0.85% sterile saline, and plated onto a solid medium. After 48 h incubation at 32 °C, total colony counts were enumerated. Time-kill curves were generated by plotting time (X-axis) against log10 transformed colony counts (Y-axis).

### 2.7. Biofilm Inhibition of CBEO Studies

The biofilm formation assay was performed according to the method described by Aiemsaard [27] with appropriate modifications. *M. furfur* in the logarithmic growth phase was adjusted to a concentration of 1 × 10^6^ CFU/mL. Different concentrations of CBEO were mixed with the fungal suspension in equal volumes and added to 12-well plates to achieve final concentrations ranging from 1/2 × MIC to 8 × MIC. After 30 h incubation at 32 °C, the culture medium was carefully aspirated, and the wells were gently washed three times with sterile PBS to remove planktonic cells. Biofilms were fixed with 10% methanol for 15 min, followed by methanol removal and air-drying. The fixed biofilms were stained with 0.1% (*w*/*v*) crystal violet for 15 min, then rinsed three times with sterile PBS until colorless and air-dried. To solubilize the dye, 33% glacial acetic acid was added, and plates were incubated at 32 °C for 20 min. Biofilm biomass was quantified by measuring absorbance at 595 nm using a microplate reader.

The biofilm formation rate of the untreated control group was normalized to 100%. Each experiment was repeated at least three times, and the average value was calculated. The percentage of biofilm formation was determined using the following formula:

Biofilm Formation (%) = (Absorbance of treated group/Absorbance of control group) × 100(2)

### 2.8. Scanning Electron Microscopy (SEM)

The effects of CBEO on the ultrastructural morphology of *M. furfur* were analyzed using scanning electron microscopy (SEM) [28]. Sterile microscopic glass slides were pre-placed in culture plates. Different concentrations of CBEO were added to *M. furfur* suspensions (1 × 10^8^ CFU/mL) and thoroughly mixed to achieve final concentrations of MIC and 2 × MIC. After 10 h incubation at 32 °C, the slides were carefully removed and gently rinsed with sterile PBS to remove residual medium and planktonic cells.

The samples were immersed in microcentrifuge tubes containing 2.5% (*v*/*v*) glutaraldehyde (electron microscopy fixative) and fixed at room temperature in the dark for 30 min, followed by overnight storage at 4 °C. Fixed specimens were washed three times (15 min each) with 0.1 M phosphate buffer (PB, pH 7.4). Subsequently, samples were post-fixed with 1% osmium tetroxide (prepared in 0.1 M PB, pH 7.4) under dark conditions at room temperature for 2 h, followed by three additional PB washes (15 min each). For dehydration, samples were sequentially treated with gradient concentrations of ethanol (15 min per step), followed by 15 min treatment with isoamyl acetate. Dehydrated samples were dried using a critical point dryer. Specimens were mounted on conductive carbon tape and sputter-coated with gold for 30 s in an ion sputter coater. Ultrastructural alterations in *M. furfur* were observed under a JEOL JSM-7610F thermal field emission scanning electron microscope.

### 2.9. Integrity of Cell Membrane Studies

Refer to WU et al. [12] to study the effect of CBEO on the permeability of *M. furfur* membrane. Logarithmic-phase *M. furfur* cultures were treated with CBEO at concentrations of 1 × MIC and 2 × MIC, followed by 10-h incubation at 32 °C. The *M. furfur* cell suspensions were subjected to a 10-min boiling water bath to serve as the positive control, ensuring complete disruption of cellular membrane integrity for optimal propidium iodide (PI) penetration and subsequent DNA binding, while untreated *M. furfur* suspensions were used as the blank control. After treatment, cells were pelleted by centrifugation at 8000× *g* for 10 min and resuspended in PBS. A 10 μM propidium iodide (PI) solution was added to the resuspended cells, which were then incubated in the dark at 37 °C for 15 min. PI fluorescence intensity internalized by *M. furfur* was quantified using a Beckman flow cytometer to analyze membrane permeability alterations. For statistical robustness, 10,000 cellular events were acquired per sample with triplicate technical replicates.

### 2.10. Determination of Cellular Content Leakage

#### 2.10.1. Leakage of Nucleotide and Protein

The leakage of nucleic acid components was assessed by measuring absorbance at 260 nm, following the method described by Chen et al. [29] with modifications. *M. furfur* cells in the logarithmic growth phase were adjusted to a concentration of 1–5 × 10^6^ CFU/mL. CBEO was added to achieve final concentrations of 1 × MIC, 2 × MIC, and FIC combinations, with PEG400-supplemented fungal suspension serving as a growth control. The cultures were incubated in a 32 °C orbital shaker at 120 rpm. Over a 12-h period, 2 mL aliquots were collected at 3-h intervals starting from 0 h, followed by centrifugation at 8000× *g* for 10 min to obtain supernatant. A standard curve was constructed using known concentrations of salmon sperm DNA standard (Sigma-Aldrich, St. Louis, MO, USA, Cat# D1626) prepared in a continuous gradient (0–300 μg/mL). Absorbance at 260 nm was measured using an ultra-micro spectrophotometer (Model NP-3000, Shanghai Esoeasy Biotechnology Co., Ltd., Shanghai, China). A sterile culture medium without fungal inoculation served as the blank control, and its absorbance was subtracted from sample readings to eliminate background interference. Filter-sterilized *M. furfur* supernatant (0.22 μm membrane filtration) was directly analyzed via spectrophotometry, with 2 μL aliquots loaded for automated nucleic acid quantification. The system calculated concentrations (μg/mL) based on A260 absorbance values and a pre-established standard curve equation.

For protein leakage analysis, sample pretreatment followed identical protocols. Protein concentrations were quantified via the bicinchoninic acid (BCA) assay. Briefly, 25 μL of standards or test samples were dispensed into a 96-well plate, mixed with 200 μL BCA working reagent, and homogenized on a plate shaker for 30 s. After 30-min incubation at 37 °C in a constant-temperature incubator, the plate was cooled to room temperature. Absorbance at 562 nm was measured using a microplate reader. Protein concentrations were calculated against a standard curve, and temporal changes in extracellular protein levels were documented.

#### 2.10.2. Leakage of Ion Measurement

The leakage of potassium (K^+^) and calcium (Ca^2+^) ions was quantified using inductively coupled plasma optical emission spectrometry (ICP-OES) [30]. *M. furfur* in the logarithmic growth phase was adjusted to a concentration of 1 × 10^6^ CFU/mL. Both 1 × MIC and 2 × MIC concentrations of CBEO were added to the fungal suspension, followed by 10 h incubation at 32 °C. The suspension was centrifuged at 8000× *g* for 10 min to collect the supernatant. The supernatant underwent microwave digestion with concentrated nitric acid. Standard solutions of K^+^ and Ca^2+^ were analyzed by ICP-OES to establish calibration curves. Free ion concentrations in the supernatant were calculated based on these curves. Results were expressed as extracellular free potassium and calcium levels in milligrams per liter of culture medium (mg/L). The experiment was carried out with three replicates per treatment and repeated twice.

### 2.11. Sorbitol Protection Assay

This experiment was based on the principle that sorbitol acts as an osmotic protectant for fungal cell walls [31]. Exogenous sorbitol was added to the liquid medium to determine the MIC of the test compound against *M. furfur*. A comparison of MIC values between sorbitol-supplemented and sorbitol-free media was performed. An increase in MIC under sorbitol supplementation suggests the cell wall may be the target site of the test compound. Specifically, CBEO’s MIC against *M. furfur* was determined in an olive oil-based medium containing 0.8 M sorbitol. Wells supplemented with 10% PEG400 solution served as negative controls. The experiment was conducted in triplicates, with results expressed as mean values from three independent replicates.

### 2.12. Ergosterol Binding Assay

Ergosterol serves as the principal sterol constituent in fungal cell membranes, playing a pivotal role in maintaining membrane stability, fluidity, and transmembrane transport functions. A microdilution assay with exogenous ergosterol supplementation was employed to assess CBEO’s MIC against *M. furfur* and compare MIC variations before and after ergosterol addition modified from Souza et al. [32], aiming to investigate whether CBEO targets fungal membrane sterols.

Ergosterol was pulverized in a mortar, dissolved in an appropriate volume of DMSO, subjected to 20 min ultrasonication in a 40 °C water bath, and cooled. Subsequently, 1% (*v*/*v*) Tween 40 was added and homogenized for 5 min. The solution was diluted to 400 μg/mL with an olive oil-based medium prior to use. Amphotericin B, processed identically, served as the positive control. Antifungal susceptibility testing via microdilution method was performed to evaluate CBEO’s MIC against *M. furfur* under 200 μg/mL exogenous ergosterol conditions. Negative controls contained 10% PEG400 solvent. Triplicate experiments were conducted, with final results calculated as mean values of three replicates.

### 2.13. Determination of Ergosterol Content by Ultra-Performance Liquid Chromatography (UPLC)

#### 2.13.1. Preparation of Standards and Determination of Wavelength

Ergosterol Quantification via Ultra-Performance Liquid Chromatography (UPLC) [33]. Ergosterol standard solutions were prepared at concentrations of 1.17, 2.34, 4.69, 9.38, 18.75, 37.5, 75, 150, and 300 μg/mL using HPLC-grade methanol. After heating and sonication to dissolve the ergosterol, solutions were diluted fivefold with methanol, filtered through 0.22 μm membranes into amber vials, and stored for UPLC analysis. Aliquots of standards were transferred via pipette, and the optimal detection wavelength was determined by full-spectrum scanning (230–300 nm) using a microvolume spectrophotometer.

#### 2.13.2. Sample Preparation for *M. furfur*

*Malassezia furfur* samples were prepared according to the method described by Gupta et al. with minor modifications [34]. Logarithmic-phase *M. furfur* cultures were transferred to sterile saline, adjusted to 1 × 10^6^ CFU/mL in m-Dixon medium, and treated with 2 × MIC, 4 × MIC, and 2 × FIC drug concentrations. Untreated fungal suspensions served as blank controls, with triplicates for each group. Cultures were incubated at 32 °C with 120 rpm shaking for 24 h. Cells were pelleted by centrifugation at 5000× *g* for 10 min, washed with sterile distilled water, and air-dried overnight at 32 °C. For ergosterol extraction, 10 mg of dried biomass was suspended in 3 mL of lysis reagent (25% KOH alcoholic solution: 25 g KOH, 35 mL distilled water, 65 mL ethanol). Samples were vortexed for 5 min and incubated at 85 °C for 1.5 h, followed by cooling to room temperature. After adding 3 mL sterile water, sterols were extracted with 9 mL n-heptane (1:3 ratio) via vigorous vortexing (5 min) and phase separation. The heptane layer was evaporated using a rotary evaporator under reduced pressure (150 rpm, 10 min) until dryness. Residues were redissolved in HPLC-grade methanol, sonicated for 10 min, filtered through 0.22 μm membranes into amber vials, and stored for UPLC analysis.

#### 2.13.3. Instrumentation Conditions

Analysis was performed on a Shimadzu Nexera LC-40 UPLC system equipped with a Shim-pack GISS C18 column (100 mm × 2.1 mm, 1.9 μm particle size). The mobile phase consisted of solvent A (methanol-glacial acetic acid, 95:5, *v*/*v*) and solvent B (methanol) under isocratic elution. Detection parameters: wavelength = 282 nm, flow rate = 0.2 mL/min, injection volume = 5 μL, column temperature = 30 °C. Chromatographic peaks were identified by retention time and spectral matching against ergosterol standards. A 20 μL aliquot was injected for each analysis. All analyses were performed in triplicate.

### 2.14. Assessment of Squalene Epoxidase (SE) Activity

Squalene, a critical intermediate in the ergosterol biosynthetic pathway, undergoes catalytic conversion by squalene epoxidase (SE) to yield 2,3-oxidosqualene, which is subsequently metabolized into ergosterol. The effect of CBEO on squalene epoxidase (SE) activity was measured using an enzyme-linked immunosorbent assay (ELISA) [35]. After culturing *M. furfur* to the logarithmic growth phase, the cells were transferred to sterile saline and diluted to 1 × 10^6^ CFU/mL using m-Dixon medium. The fungal suspensions were treated with 1 × MIC and 2 × MIC concentrations of CBEO, with untreated suspensions serving as blank controls. Triplicate samples were prepared for each treatment group. The cultures were incubated at 32 °C with 120 rpm shaking for 24 h. Subsequently, the suspensions were transferred to pre-chilled conical centrifuge tubes and centrifuged at 8000× *g* to pellet cells. The cell pellets were resuspended in 1 mL phosphate-buffered saline (PBS, pH 7.2–7.4, 0.01 M). The enzymatic activity of fungal squalene epoxidase (SE) was quantitatively analyzed using a colorimetric method according to the manufacturer’s protocol of an ELISA-based squalene epoxidase activity detection kit.

### 2.15. Statistical Analysis

Quantitative data were presented as the mean ± standard deviation (SD). The IBM SPSS Statistics 27 (SPSS Inc. Chicago, IL, USA) was employed for one-way ANOVA analysis followed by the LSD test for multiple comparisons when the variance is homogeneous, if not, by Tamhane’s T2 (M) and Dunnett’s T3 test. *p* Levels of significance were set at *p* < 0.05. GraphPad Prism 10.1.2 software (GraphPad Software, San Diego, CA, USA) was used for plotting.

## 3. Results

### 3.1. Phytochemical Characterization by GC-MS Analysis

**Figure 3:**
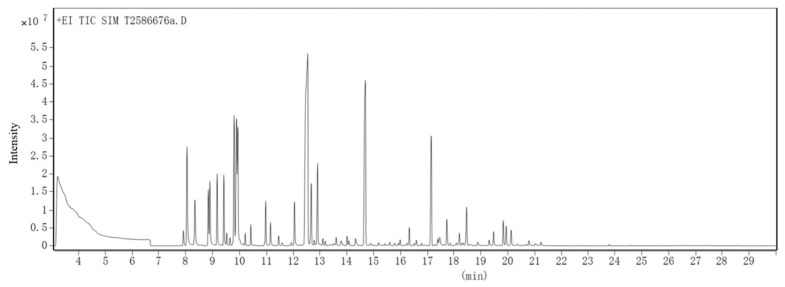
GC-MS total ion chromatogram (TIC) of CBEO.

The composition of CBEO was analyzed by gas chromatography-mass spectrometry (GC-MS) devices. A quantitative analysis was carried out using peak area normalization measurements without correction factors as percentages of each component, as shown in Figure 3 and Table 1. Anyway, the high diversity in chemical compositions was marked for CBEO, and the identified compounds included 6 types of compounds, such as -hydrin, -ester, -alkene, -aldehyde, -ketone, and -amine, etc. In this assay, the relative content of nineteen compositions exceeded 1% in the identified chemical components, with a predominance of D-borneol, which accounted for 27.84% of the total composition. In addition, the components with higher relative content were Benzene, 1-methyl-3-(1-methylethyl)- (8.90%), α-Terpineol (7.23%), Bicyclo [2.2.1] heptan-2-ol, 1,7,7-trimethyl-, acetate, (1S-endo)- (7.16%), α-Pinene (4.60%) and D-Limonene (3.59%) etc.

### 3.2. Thermal Stability Analysis of CBEO

**Figure 4:**
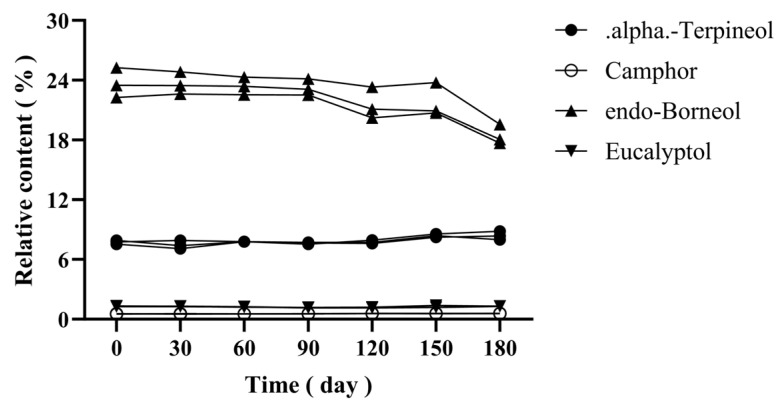
Stability of four characteristic components in three batches of CBEO.

All three batches exhibited exceptional stability during 180-day storage at 40 °C, as shown in Figure 4, with Relative Standard Deviations (RSD) values for the four characteristic components (α-terpineol, camphor, D-borneol, and eucalyptol) within each batch during storage, as well as the inter-batch RSD values of these components across three batches at each testing interval, were consistently below 10%. This confirms CBEO’s chemical robustness under prolonged thermal stress, validating its suitability for standardized antifungal formulations.

### 3.3. Determination of MIC, MFC and FICI Values

**Figure 5:**
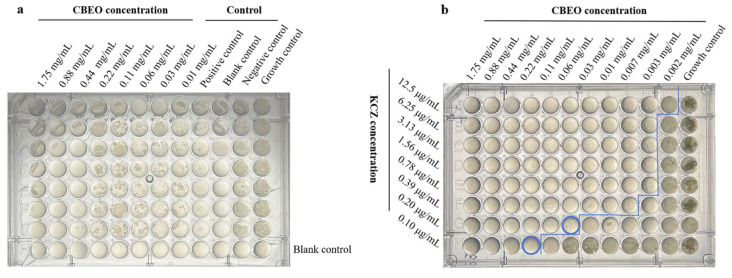
(**a**) MIC determination results of CBEO against *M. furfur*. (**b**) Synergistic Antibacterial Results (FIC) of CBEO Combined with Antifungal Agent KCZ. The blue line segments indicate the demarcation of bacterial colony growth, and the blue circles mark the potential minimum combined antibacterial index wells.

The antifungal activity of CBEO was determined by assessing its minimum inhibitory concentration (MIC) and minimum fungicidal concentration (MFC). Notably, experimental results from Table 2 and Figure 5a revealed that CBEO exhibited an MIC value of 0.88 mg/mL against *M. furfur*, with further confirmation via the dilution plating method yielding an MFC value of 1.75 mg/mL, demonstrating potent antifungal efficacy against *M. furfur*. Checkerboard microdilution assays demonstrated a fractional inhibitory concentration index (FICI) of 0.5 for the combination of 0.22 mg/mL CBEO with 0.10 μg/mL ketoconazole, indicating a synergistic interaction between the two compounds (Figure 5b).

### 3.4. Time-Kill Kinetic Curve Analysis

**Figure 6:**
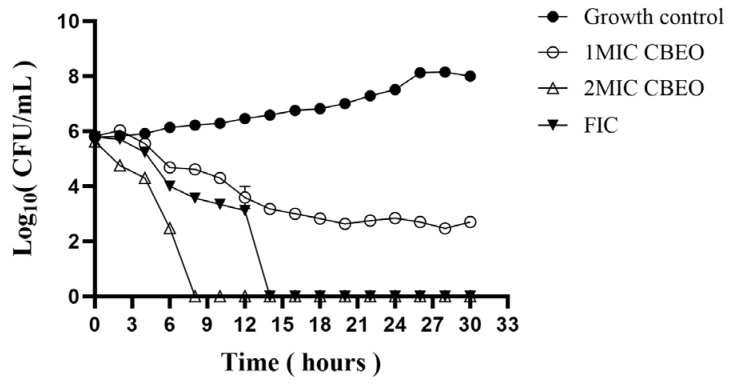
Time-Killing Kinetics Curve.

Time-kill kinetic curves were plotted to evaluate the bactericidal effects of CBEO at varying concentrations against *M. furfur* and to elucidate its antimicrobial mode of action. As shown in Figure 6, the growth control group exhibited four distinct microbial growth phases: the culture entered the logarithmic phase at 20 h, reached the stationary phase by 26 h, and initiated the death phase at 30 h.

Under 1 × MIC CBEO treatment, fungal proliferation was significantly inhibited, with viable cell counts gradually declining over time and stabilizing after 20 h. At 2 × MIC CBEO, a sharp reduction in viable cells occurred within 0–8 h, followed by complete growth suppression with no further changes. The fractional inhibitory concentration (FIC) combination eradicated all viable *M. furfur* cells within 0–14 h of exposure. These results demonstrate a clear time- and dose-dependent antimicrobial pattern of CBEO. Higher CBEO concentrations prolonged both the lag phase and the time required for the fungal suspension to reach maximum cell density.

### 3.5. Impact of CBEO on Biofilm Biomass of M. furfur

**Figure 7:**
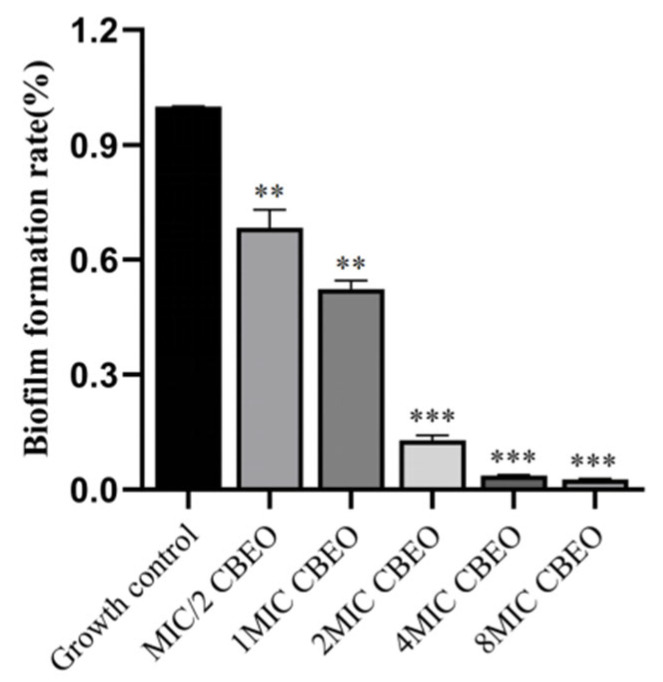
*M. furfur* biofilm biomass statistical results. At the 95% confidence interval, compared with the growth control group, ** indicates a highly significant difference (*p* < 0.01); *** indicates an extremely significant difference (*p* < 0.001).

This study demonstrated a concentration-dependent reduction in biofilm area and significant decreases in viable cell ratios following CBEO treatment. As illustrated in Table 3 and Figure 7, biofilm clearance rates reached 32% ± 15% and 48% ± 7% for 1/2 × MIC and 1 × MIC CBEO treatments, respectively. Compared to the growth control, treated biofilms exhibited reduced coverage with fragmented, irregular morphologies, indicating structural disruption. At 2 × MIC CBEO, biofilm clearance escalated to 87% ± 4%, accompanied by near-complete biofilm eradication, demonstrating potent inhibitory effects on *M. furfur* biofilm formation.

### 3.6. Scanning Electron Microscopy (SEM) Analysis

**Figure 8:**
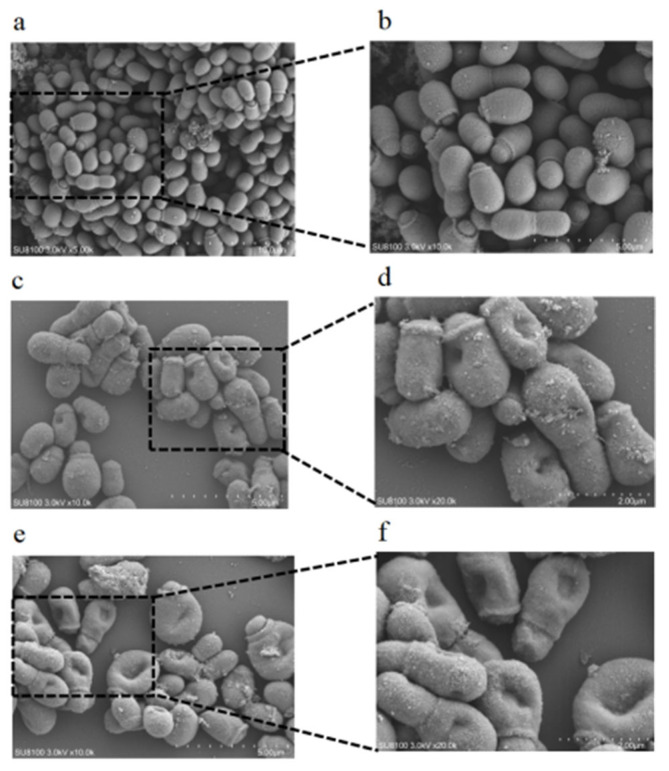
SEM results of *M. furfur* exposed to different concentrations of CBEO. (**a**) magnification of 5.00 k×, (**b**,**c**,**e**) magnification of 10.0 k×, (**d**,**f**) magnification of 20.0 k×; (**a**,**b**) are untreated control group, (**c**,**d**) are intervention group with MIC concentration of CBEO, (**e**,**f**) are intervention group with 2 × MIC concentration of CBEO.

This study investigated the ultrastructural alterations in *M. furfur* following CBEO intervention. Scanning electron microscopy (SEM) revealed that untreated control cells exhibited oval-shaped budding cells in various growth stages, with intact and smooth surfaces, clustered morphology, and coverage by an extracellular polymeric substance (EPS) matrix. In contrast, *M. furfur* treated with MIC CBEO displayed significant morphological aberrations: surface roughness, localized pore formation, and partial cellular shrinkage with irregular contours. At 2 × MIC CBEO, severe structural collapse was observed, including membrane invagination, cellular rupture, and sporangiophore fragmentation (Figure 8). These findings demonstrate that CBEO induces membrane poration, leading to osmotic imbalance and lethal dehydration. The resultant cell lysis explains the high proportion of membrane-damaged cells in CBEO-treated suspensions.

### 3.7. Permeability of Cell Membrane Studies

**Figure 9:**
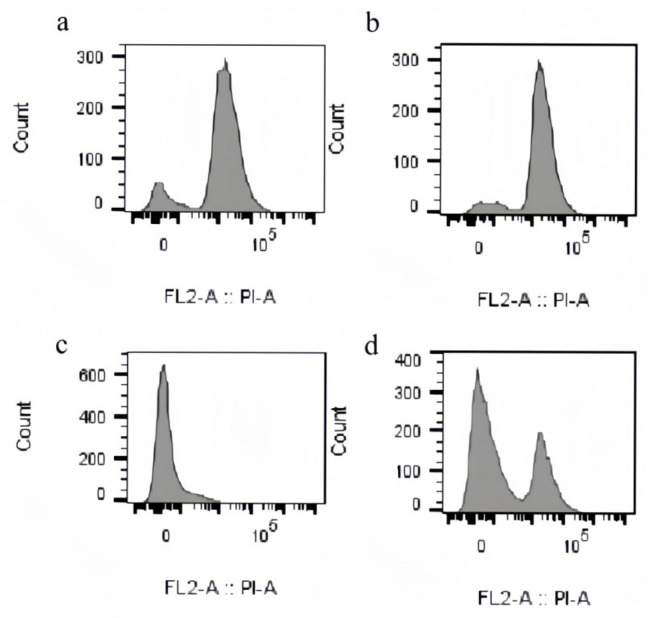
Permeability determination of *M. furfur* cell membrane. (**a**) is the blank control, (**b**) is the 1 × MIC CBEO group, (**c**) is the 2 × MIC CBEO group, and (**d**) is the positive control group.

Disruption of fungal cell membrane integrity represents a common antifungal mechanism. Flow cytometric analysis was employed to assess CBEO-induced membrane damage in *M. furfur*. This experiment utilized propidium iodide (PI), a fluorescent dye that binds to intracellular DNA, to evaluate alterations in cell membrane permeability under varying CBEO concentrations. As depicted in Figure 9a–d, compared to the growth control, treatment with 1 × MIC and 2 × MIC CBEO significantly enhanced fluorescence intensity in *M. furfur* cells. Furthermore, membrane permeability increased proportionally with CBEO concentration, demonstrating a distinct dose-dependent effect.

### 3.8. Membrane Integrity Analysis

#### 3.8.1. Analysis of Nucleic Acid and Protein Leakage

**Figure 10:**
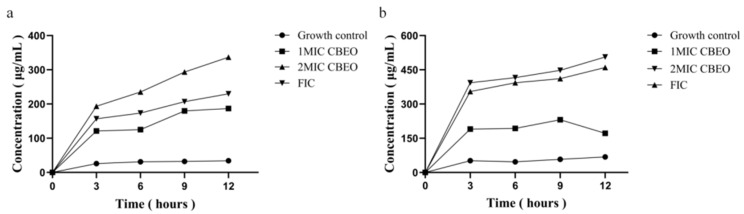
Intracellular nucleic acid and protein leakage in *M. furfur*. (**a**) extracellular nucleic acid leakage. (**b**) extracellular protein leakage.

As shown in Figure 10a,b, significant increases in extracellular nucleic acid (detected via UV spectrophotometry at 260 nm) and protein levels (measured by BCA assay) were observed in CBEO-treated supernatants. Compared to the growth control, escalating concentrations and prolonged treatment durations progressively elevated nucleic acid and protein release from *M. furfur* cells, further confirming the membrane-damaging effects of CBEO.

#### 3.8.2. Quantification of Potassium and Calcium Ion Efflux

**Figure 11:**
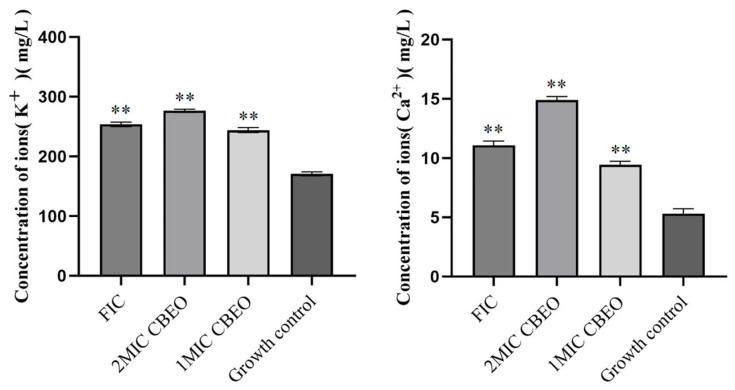
Intracellular potassium and calcium ion leakage in *M. furfur*. At the 95% confidence interval, compared with the growth control group, ** indicates a highly significant difference (*p* < 0.01).

As shown in Figure 11, the mean K^+^ concentrations in the supernatant increased sequentially from 170.89 ± 3.26 mg/L to 244.02 ± 4.35 mg/L, 276.52 ± 2.64 mg/L and 253.70 ± 3.77 mg/L. When treated with 2 × MIC CBEO, the Ca^2+^ concentration in the supernatant rose from 5.30 ± 0.41 mg/L to 14.89 ± 0.58 mg/L. Given the critical roles of K^+^ in cytoplasmic pH regulation and cellular architecture maintenance, coupled with Ca^2+^’s influence on diverse physiological activities, the efflux of these ions substantiates CBEO-induced membrane rupture via osmotic dysregulation. The leakage of macromolecules and ions from *M. furfur* cells likely results from CBEO-mediated increases in membrane permeability, which severely compromises membrane integrity. This mechanism represents a critical factor in the CBEO-triggered apoptosis of *M. furfur*.

### 3.9. Effect of CBEO on the Cell Membrane/Wall Structure of M. furfur

As shown in Table 4, the minimum inhibitory concentration (MIC) of CBEO against *M. furfur* remained unchanged in media with or without exogenous sorbitol supplementation, suggesting that the cell wall is unlikely to be the primary target of CBEO. However, CBEO’s antifungal efficacy was significantly influenced by extracellular ergosterol. Under exogenous ergosterol conditions, the MIC value increased 8-fold compared to the non-supplemented group. Amphotericin B, a well-characterized ergosterol-targeting agent used as a positive control, exhibited a 16-fold elevation in MIC against *M. furfur*.

### 3.10. Analytical Determination of Ergosterol Content

**Figure 12:**
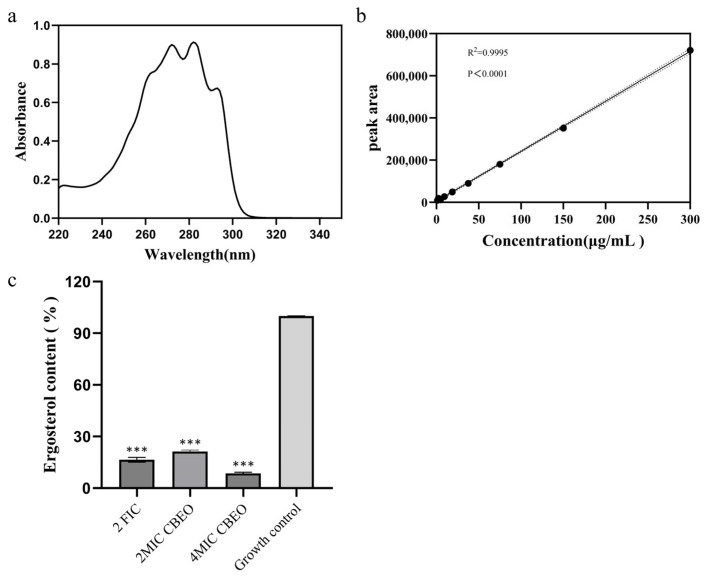
Ergosterol content in *M. furfur* treated with CBEO. (**a**) displays the ultraviolet absorption spectrum of ergosterol. (**b**) illustrates the ergosterol standard calibration curve. (**c**) is statistics of the determination results of ergosterol content. At the 95% confidence interval, compared with the growth control group, *** indicates an extremely significant difference (*p* < 0.001).

This study investigated ergosterol content alterations in *M. furfur* following CBEO treatment. Full-wavelength UV absorption scanning identified a characteristic ergosterol absorption peak at 282 nm (Figure 12a: UV absorption spectrum. The results exhibited a significantly characteristic four-peak curve of ergosterol). As shown in Figure 12b, the peak area of ergosterol standard solutions exhibited excellent linearity (R^2^ = 0.9995) within the 1.17–300 μg/mL concentration range, with a regression equation of Y = 2373X + 4295.

Ergosterol content in test samples was quantified by substituting measured peak areas into the standard curve. Figure 12c presents the relative percentage content of ergosterol, which demonstrates that untreated *M. furfur* (growth control) yielded the highest ergosterol content (113.63 ± 0.28 μg/mL), while the high-dose CBEO group (2 × MIC) showed the lowest levels (9.06 ± 0.13 μg/mL). Ergosterol content progressively decreased with increasing CBEO concentrations, exhibiting a dose-dependent effect (*p* < 0.001 vs. control). Notably, 2 × MIC, 4 × MIC, and 2 × FIC CBEO treatments demonstrated highly significant inhibitory effects on *M. furfur* membrane ergosterol biosynthesis.

### 3.11. Analytical Determination of Squalene Epoxidase (SE) Content

**Figure 13:**
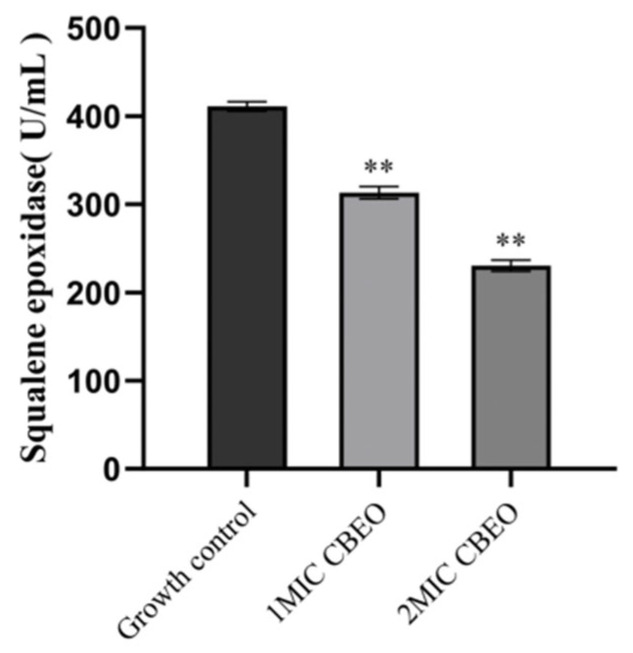
Squalene epoxidase level. At the 95% confidence interval, compared with the growth control group, ** indicates a highly significant difference (*p* < 0.01).

Following treatment with 1 × MIC and 2 × MIC CBEO, the squalene epoxidase (SE) activity in *M. furfur* cultures was measured as 313.15 ± 6.92 U/L and 230.41 ± 6.26 U/L, respectively. In contrast, the untreated growth control group exhibited significantly higher SE activity (411.07 ± 5.16 U/L). Statistical analysis revealed highly significant differences between both 1 × MIC and 2 × MIC CBEO treatment groups compared to the untreated control (*p* < 0.01). The statistical outcomes are graphically summarized in Figure 13.

## 4. Discussion

This study identified 78 bioactive constituents in Cinnamomum burmannii essential oil (CBEO) through gas chromatography-mass spectrometry (GC-MS) analysis, with D-borneol (27.8%) as the signature component, suggesting potential multi-target effects due to its multi-component nature. In view of the volatile nature of CBEO, this study evaluated the thermal stability of CBEO by monitoring the dynamic variations of four characteristic constituents. Experimental data revealed that three independently produced batches of CBEO maintained consistent chemical profiles under accelerated storage conditions at 40 °C for 180 days.

Given the absence of standardized protocols for *Malassezia* spp. antifungal susceptibility testing—neither CLSI nor EUCAST has issued unified guidelines [36]—this study employed modified CLSI broth microdilution (BMD) assays in olive oil-based medium (simulating skin lipid environments) to evaluate CBEO’s efficacy [37,38,39]. CBEO exhibited potent inhibitory activity against *M. furfur*, with MIC = 0.88 mg/mL and MFC = 1.75 mg/mL. Synergistic interaction with ketoconazole (FICI = 0.5) suggests CBEO’s potential as a natural antifungal adjuvant in cosmetic formulations, enhancing efficacy while mitigating toxicity risks associated with prolonged monotherapy.

Biofilms enhance microbial pathogenicity toward hosts and confer resistance to antimicrobial agents such as antibiotics. Studies have revealed that *M. furfur* also possesses biofilm-forming capability, which facilitates its evasion of antifungal interventions. CBEO’s chemical complexity underpins its multi-modal antimicrobial mechanisms. Time- and dose-dependent inhibition of *M. furfur* growth was observed, with biofilm formation suppressed by 87% ± 4% at 2 × MIC CBEO, outperforming the 4 × MIC requirement for geraniol reported by Gupta et al. [34] against Candida albicans biofilms, indicating enhanced antibiofilm activity through component synergy. The fungal cell membrane is embedded with diverse proteins and glycans that mediate essential metabolic transport functions. Membrane disruption leads to leakage of intracellular macromolecules such as nucleic acids and proteins, ultimately causing cell death. Therefore, quantifying nucleic acid and protein concentrations in fungal suspensions serves as a critical indicator of membrane integrity impairment. Mechanistic investigations revealed a direct correlation between CBEO-induced K^+^/Ca^2+^ efflux and increased membrane permeability. Building upon Chen et al.’s [29] theory of ion imbalance-mediated disruption of intracellular pH homeostasis.

The fungal cell wall and cell membrane protect cellular integrity against external stressors, safeguarding organelles and cytoplasmic constituents. Ergosterol, the principal sterol component of fungal cell membranes, constitutes approximately 10% of fungal dry weight. It stabilizes membrane architecture by interacting with phospholipids and modulates membrane fluidity, thereby maintaining structural integrity, cellular viability, and material transport. This molecule serves as a key therapeutic target for most clinically available antifungals [40,41]. Although both ketoconazole (KCZ) and CBEO target the ergosterol biosynthesis pathway in fungal cell membranes, their mechanisms of action exhibit fundamental differences. As a representative azole antifungal, KCZ primarily inhibits cytochrome P450-dependent 14α-demethylase (CYP51), thereby blocking the conversion of ergosterol to lanosterol and leading to toxic sterol intermediate accumulation—a process frequently associated with CYP51 mutations or efflux pump overexpression. In contrast, our findings demonstrate that CBEO not only suppresses squalene epoxidase (SE) activity to disrupt ergosterol synthesis but also contains monoterpenes that physically destabilize the lipid bilayer architecture of fungal membranes [42]. This dual action induces membrane permeability elevation, ion gradient collapse, and cytoplasmic leakage, establishing a multi-target assault paradigm distinct from KCZ’s single-target inhibition. CBEO’s complex composition may mitigate resistance risks through mechanisms such as efflux pump inhibition or interference with resistance-related signaling pathways [43], though further validation is required. Scanning electron microscopy (SEM) revealed membrane integrity destruction in CBEO-treated *M. furfur*, manifested as surface pitting and structural collapse. Besides, UPLC analysis revealed a significant reduction in ergosterol content under CBEO treatment. This study further reveals that CBEO suppresses squalene epoxidase (SE) activity to block ergosterol biosynthesis, a mechanism distinct from the classical membrane sterol-targeting pathway of amphotericin B. Such divergence may reduce drug resistance risks and establish a “membrane damage–ion dyshomeostasis–metabolic suppression” cascade effect. This multi-target mechanism likely impedes the development of antimicrobial resistance.

## 5. Conclusions

This study is the first to elucidate the antifungal activity and mechanisms of steam-distilled CBEO (post-borneol crystallization) against *M. furfur*. In conclusion, CBEO exerts its antifungal effects through a tripartite cascade mechanism encompassing membrane disruption, ionic disequilibrium, and inhibition of enzymatic metabolism. The multi-component nature of CBEO not only potentiates its antifungal efficacy but also likely mitigates resistance development by polypharmacological targeting, providing a scientific foundation for designing novel combinatorial antifungal formulations. Future investigations should focus on isolating individual CBEO constituents and quantifying their synergistic interactions through isobolographic analysis, thereby optimizing its pharmacotherapeutic potential against evolving fungal resistance mechanisms.

## Figures and Tables

**Table 1:** Compositions of volatile components from CBEO. Values are averages of three parallel measurements.

Compounds	NIST_RI	CAS	Relative Content (%)
endo-Borneol	1170	507-70-0	27.84
Benzene, 1-methyl-3-(1-methylethyl)-	1022	535-77-3	8.90
.alpha.-Terpineol	1196	98-55-5	7.23
Bicyclo[2.2.1]heptan-2-ol, 1,7,7-trimethyl-, acetate, (1S-endo)-	1287	5655-61-8	7.16
.alpha.-Pinene	936	80-56-8	4.60
D-Limonene	1031	5989-27-5	3.59
Dihydrocarvyl acetate	1306	20,777-49-5	3.59
.beta.-Ocimene	1037	13,877-91-3	3.27
.alpha.-Phellandrene	1005	99-83-2	3.14
2-Pentanol	699	6032-29-7	2.09
Cedrene	1422	11,028-42-5	1.73
.beta.-Myrcene	992	123-35-3	1.40
Pentanoic acid, 2-hydroxy-3-methyl-, methyl ester	989	41,654-19-7	1.38
Eucalyptol	1034	470-82-6	1.37
Levomenthol	1177	2216-51-5	1.37
Nonane, 4,5-dimethyl-	1046	17,302-23-7	1.35
d-Menthol	1167	15,356-60-2	1.32
Pantolactone	1037	599-04-2	1.16
4-Nonanone	1030	4485-09-0	1.16
1H-Cyclopropa[a]naphthalene, 1a,2,3,5,6,7,7a,7b-octahydro-1,1,7,7a-tetramethyl-, [1aR-(1a.alpha.,7.alpha.,7a.alpha.,7b.alpha.)]-	1432	17,334-55-3	0.87
(3R,3aR,7R,8aS)-3,8,8-Trimethyl-6-methyleneoctahydro-1H-3a,7-methanoazulene	1414	79,120-98-2	0.82
Cyclohexene, 1-methyl-4-(1-methylethylidene)-	1091	586-62-9	0.79
Butanoic acid, 3-methyl-, 1-ethenyl-1,5-dimethyl-4-hexenyl ester	1464	1118-27-0	0.77
Butanoic acid, 3-methyl-3-nitroso-, methyl ester	1019	49,680-44-6	0.58
Camphor	1151	76-22-2	0.54
Geranyl formate	1301	105-86-2	0.54
cis-Chrysanthenol	1162	55,722-60-6	0.51
1-Tridecene	1292	2437-56-1	0.42
6-Undecanol	1281	23,708-56-7	0.42
Camphenone, 6-	1095	55,659-42-2	0.38
5-Nonenal, (E)-	1107	2277-18-1	0.38
.beta.-Bisabolene	1509	495-61-4	0.38
Bicyclo[4.1.0]hept-2-ene, 3,7,7-trimethyl-, (1S-cis)-	985	4497-92-1	0.37
Camphene	952	79-92-5	0.36
Cyclopentanone, 2-methyl-3-(1-methylethyl)-	1174	54,549-81-4	0.35
Cyclohexanepropanoic acid, 2-propenyl ester	1435	2705-87-5	0.33
trans-.beta.-Ocimene	1049	3779-61-1	0.31
Bicyclo[3.1.0]hexan-2-ol, 2-methyl-5-(1-methylethyl)-, (1.alpha.,2.beta.,5.alpha.)-	1070	15,537-55-0	0.31
2H-2a,7-Methanoazuleno[5,6-b]oxirene, octahydro-3,6,6,7a-tetramethyl-	1585	29,597-36-2	0.30
Methacrylamide	1149	79-39-0	0.30
Citral	1273	5392-40-5	0.26
2-Decanone	1193	693-54-9	0.26
2H-Pyran-2-one, tetrahydro-6-propyl-	1288	698-76-0	0.26
Humulene	1467	6753-98-6	0.25
7-Oxabicyclo[4.1.0]heptane, 1-methyl-4-(2-methyloxiranyl)-	1294	96-08-2	0.25
5-Methylhexanoic acid	1043	628-46-6	0.25
5-Octen-1-ol, (Z)-	1074	64,275-73-6	0.25
N,N′-Methylenebismethacrylamide	1566	2359-15-1	0.23
Isobornyl formate	1233	1200-67-5	0.23
Acetic acid, cinnamyl ester	1446	103-54-8	0.21
Nerolidol	1544	142-50-7	0.21
Bicyclo[3.1.1]hept-3-en-2-one, 4,6,6-trimethyl-, (1S)-	1204	1196-01-6	0.20
.beta.-Pinene	980	127-91-3	0.20
1H-Cycloprop[e]azulen-7-ol, decahydro-1,1,7-trimethyl-4-methylene-, [1ar-(1a.alpha.,4a.alpha.,7.beta.,7a.beta.,7b.alpha.)]-	1576	6750-60-3	0.18
Geranyl acetate	1384	105-87-3	0.18
Cinnamaldehyde, (E)-	1270	14,371-10-9	0.18
Niacinamide	1419	98-92-0	0.17
Aromandendrene	1440	489-39-4	0.17
p-Mentha-1,8-dien-7-ol	1297	536-59-4	0.16
D-Fenchone	1103	4695-62-9	0.15
3,3-Dimethyl-6-methylenecyclohexene	1001	20,185-16-4	0.15
.gamma.-Elemene	1433	29,873-99-2	0.15
Alloaromadendrene	1461	25,246-27-9	0.15
Cedrol	1600	77-53-2	0.13
Succinic anhydride	1023	108-30-5	0.13
3-Buten-2-ol, 4-(2,6,6-trimethyl-1-cyclohexen-1-yl)-	1428	22,029-76-1	0.13
2-Ethylhexyl methacrylate	1296	688-84-6	0.12
1,3-Cyclohexadiene, 5-(1,5-dimethyl-4-hexenyl)-2-methyl-, [S-(R*,S*)]-	1495	495-60-3	0.11
1-Butanamine, 3-methyl-N-(3-methylbutylidene)-	1047	35,448-31-8	0.11
.beta.-Guaiene	1490	88-84-6	0.11
2,6,6-Trimethyl-2-cyclohexene-1,4-dione	1147	1125-21-9	0.10
Thymol	1291	89-83-8	0.10
4aH-Cycloprop[e]azulen-4a-ol, decahydro-1,1,4,7-tetramethyl-, [1aR-(1a.alpha.,4.beta.,4a.beta.,7.alpha.,7a.beta.,7b.alpha.)]-	1568	5986-49-2	0.10
(+)-4-Carene	1009	29,050-33-7	0.10
Eugenol	1362	97-53-0	0.10
δ-cadinol	1610	36,564-42-8	0.10
Salvial-4(14)-en-1-one	1595	73,809-82-2	0.09
2,6-Octadien-1-ol, 3,7-dimethyl-, acetate, (Z)-	1365	141-12-8	0.09
Total			100

**Table 2:** CBEO’s Antifungal Activity Determination against *M. furfur* (mg/mL). “+” indicates fungal growth in the well. “−” indicates no fungal growth in the well. Positive control: Ketoconazole (KCZ). Negative control: Solvent control (10% PEG400). Growth control: Untreated fungal suspension. Blank control: Sterile liquid medium (no fungal inoculation).

Strain	Concentration of CBEO					Positive Control	Negative Control	Growth Control	Blank Control
	**1.75**	**0.88**	**0.44**	**0.22**	**0.11**				
*M. furfur* ATCC44344	−	−	+	+	+	−	+	+	−

**Table 3:** Amount of biofilm formation under different concentrations of CBEO intervention ($\bar{x}\pm SD$).

$\mathrm{Biofilm}\;\mathrm{Formation}\;\mathrm{of}\;M.\;\mathrm{furfur}\;(\bar{x}\pm SD$ **)**					
Growth Control	1/2 × MIC CBEO	1 × MIC CBEO	2 × MIC CBEO	4 × MIC CBEO	8 × MIC CBEO
1.00	0.68 ± 0.15	0.52 ± 0.07	0.13 ± 0.04	0.04	0.03

**Table 4:** Effect of CBEO on MIC value of *M. furfur* ($\bar{x}\pm SD$). “−” data is not measured or has no drug concentration, “+” data indicates drug existence; Within a 95% confidence interval, ** denotes highly significant differences (*p* < 0.01).

Groups	Effect of CBEO on MIC Value of *M. furfur* (mg/mL)			
	Sorbitol (0.8 M)		Ergosterol	
	−	+	−	+
CBEO	0.875	0.875	0.875	7.000 **
AMB	−	−	0.008	0.128 **

## Data Availability

The original contributions presented in the study are included in the article; further inquiries can be directed to the corresponding author.
